# Neurocognitive and functional impairment in adult and paediatric tuberculous meningitis

**DOI:** 10.12688/wellcomeopenres.15516.1

**Published:** 2019-11-13

**Authors:** Angharad G. Davis, Sam Nightingale, Priscilla E. Springer, Regan Solomons, Ana Arenivas, Robert J. Wilkinson, Suzanne T. Anderson, Felicia C. Chow, Tuberculous Meningitis International Research Consortium, Rob E. Aarnoutse, Rob E. Aarnoutse, Suzanne T. B. Anderson, Suzanne T. B. Anderson, Nathan C. Bahr, Nguyen D. Bang, David R. Boulware, Tom Boyles, Lindsey H. M. te Brake, Satish Chandra, Felicia C. Chow, Fiona V. Cresswell, Reinout van Crevel, Angharad G. Davis, Sofiati Dian, Joseph Donovan, Kelly E. Dooley, Anthony Figaji, A. Rizal Ganiem, Ravindra Kumar Garg, Diana M. Gibb, Raph L. Hamers, Nguyen T. T. Hiep, Darma Imran, Akhmad Imron, Sanjay K. Jain, Sunil K. Jain, Byramee Jeejeebhoy, Jayantee Kalita, Rashmi Kumar, Vinod Kumar, Arjan van Laarhoven, Rachel P-J. Lai, Abi Manesh, Suzaan Marais, Vidya Mave, Graeme Meintjes, David B. Meya, Usha K. Misra, Manish Modi, Alvaro A. Ordonez, Nguyen H. Phu, Sunil Pradhan, Kameshwar Prasad, Alize M. Proust, Lalita Ramakrishnan, Ursula Rohlwink, Rovina Ruslami, Johannes F. Schoeman, James A. Seddon, Kusum Sharma, Omar Siddiqi, Regan S. Solomons, Nguyen T. T. Thuong, Guy E. Thwaites, Ronald van Toorn, Elizabeth W. Tucker, Sean A. Wasserman, Robert J. Wilkinson

**Affiliations:** 1 University College London, Gower Street, London, WC1E 6BT, UK; 2 Francis Crick Institute, Midland Road, London, NW1 1AT, UK; 3 Institute of Infectious Diseases and Molecular Medicine. Department of Medicine, University of Cape Town, Observatory, 7925, South Africa; 4 HIV Mental Health Research Unit, University of Cape Town,, Observatory, 7925, South Africa; 5 Department of Paediatrics and Child Health, Faculty of Medicine and Health Sciences, Stellenbosch University, Cape Town, South Africa; 6 The Institute for Rehabilitation and Research Memorial Hermann, Department of Rehabilitation Psychology and Neuropsychology,, Houston, Texas, USA; 7 Baylor College of Medicine, Department of Physical Medicine and Rehabilitation, Houston, Texas, USA; 8 Department of Infectious Diseases, Imperial College London, London, W2 1PG, UK; 9 Wellcome Centre for Infectious Disease Research in Africa, Institute of Infectious Diseases and Molecular Medicine at Department of Medicine, University of Cape Town, Observatory, 7925, South Africa; 10 MRC Clinical Trials Unit at UCL, University College London, London, WC1E 6BT, UK; 11 Evelina Community, Guys and St Thomas’ NHS Trust, 5 Dugard Way, London, SE11 4TH, UK; 12 Weill Institute of Neurosciences, Department of Neurology and Division of Infectious Diseases, University of California, San Francisco, California, USA

**Keywords:** Tuberculous Meningitis, Neurocognitive, Functional, Neurobehavioural, Neurodevelopmental, Psychiatric

## Abstract

In those who survive tuberculous meningitis (TBM), the long-term outcome is uncertain; individuals may suffer neurocognitive, functional and psychiatric impairment, which may significantly affect their ability to lead their lives as they did prior to their diagnosis of TBM. In children who survive, severe illness has occurred at a crucial timepoint in their development, which can lead to behavioural and cognitive delay. The extent and nature of this impairment is poorly understood, particularly in adults. This is in part due to a lack of observational studies in this area but also inconsistent inclusion of outcome measures which can quantify these deficits in clinical studies. This leads to a paucity of appropriate rehabilitative therapies available for these individuals and their caregivers, as well as burden at a socioeconomic level. In this review, we discuss what is known about neurocognitive impairment in TBM, draw on lessons learnt from other neurological infections and discuss currently available and emerging tools to evaluate function and cognition and their value in TBM. We make recommendations on which measures should be used at what timepoints to assess for impairment, with a view to optimising and standardising assessment of neurocognitive and functional impairment in TBM research.

## Introduction

Neurocognitive and functional impairment is a long-term complication of tuberculous meningitis (TBM); however, data on physical, cognitive, and psychiatric sequelae of TBM, which have lasting socioeconomic implications for patients and their families, are limited. Detailed characterization of neurocognitive and functional impairment is critical to research aimed at improving prediction of recovery in TBM and optimization of rehabilitation after acute illness. In addition, cognitive and functional outcome measures can serve as trial endpoints that have reasonable properties for statistical analysis and are clinically meaningful to patients and their families. By standardizing the instruments and battery of tests used to assess these outcomes in TBM and ensuring that they are appropriate for use in diverse cultural and multinational settings, data can be pooled and compared across studies to maximize trial efficiency. Furthermore, a better understanding of the long-term clinical manifestations of TBM, including the characterisation and timeframe of brain injury, will guide the study of pathogenic mechanisms in TBM. In turn, this will enable the development of novel biomarkers and therapeutic interventions to risk stratify and treat neurocognitive impairment in TBM.

## What is known about neurocognitive impairment in TBM?

### Adult

Few published studies have described neurocognitive outcomes in adult TBM. A retrospective New Zealand study detected cognitive impairment in 12% of adult TBM survivors at a median follow-up of 18 months (range 1–197 months), although how cognition was assessed is unclear ^1^. In two cohort studies from India (n=30 and n=65), patients were evaluated with the 30-point Mini Mental State Exam (MMSE) ^2^ at six months ^3^ and one year ^4^ after TBM diagnosis. Using cut-off scores of 22 to 29, depending on education level, over half of patients (54% and 55%, respectively) were impaired. Only one study in Taiwan has used more in-depth neuropsychological testing to quantify neurocognitive impairment in 17 adult TBM survivors compared with controls, with deficits present in several domains, including speed of information processing and working memory ^5^.

The pathogenesis of neurocognitive impairment in TBM is unclear but likely multifactorial. Potential mechanisms include cerebrovascular complications of TBM and hydrocephalus. When the basal inflammatory process extends into the parenchyma, encephalitis may occur ^6^. Recent studies utilising diffusion tensor imaging ^7^ and voxel-based morphometric MRI ^5^ have demonstrated white and grey matter abnormalities associated with worse neuropsychological outcomes in patients with TBM.

Clinically, stroke in TBM occurs in up to 20% of patients ^8^. Computed tomography (CT) and magnetic resonance imaging (MRI) however, may reveal cerebral infarctions in a larger proportion of patients (35% and 57% respectively) ^9^. The most common location for infarction is within the ‘tubercular zone’ encompassing the caudate head, anteriomedial thalami, and anterior limb and genu of the internal capsule ^10^. Patients with clinically ‘silent’ infarcts may present with neurocognitive and functional impairment even in the absence of occult physical disability.

In addition to cerebrovascular changes, hydrocephalus secondary to TBM affects cognition and is associated with worse outcomes and higher risk of stroke ^11^. Acutely, hydrocephalus presents with reduced level of consciousness and seizures; however, its effect on long-term neurocognitive outcomes is unknown. Adults with normal pressure hydrocephalus may be impaired in multiple cognitive domains, including memory, learning, psychomotor and executive function. Likewise, chronic hydrocephalus post-TBM may cause similar dysfunction ^12^.

### Paediatric

By contrast to adult TBM, there is a greater body of knowledge describing neurodevelopmental outcomes following childhood TBM. The largest burden of tuberculosis (TB), and therefore childhood TBM, is borne by low and middle income countries (LMIC) ^13^. TBM causes long-term cognitive, motor, language, and behavioural sequelae ^14– 17^. A meta-analysis on treatment outcomes in childhood TBM showed that the risk of neurological sequelae was 54% among survivors ^18^.

Most long-term outcome studies (>2 years after completion of treatment) were carried out in the decades following the advent of chemotherapy ^19– 22^. In a more recent follow-up study of childhood TBM survivors, the most common impairments were in cognition, learning, emotion, and behaviour, all potentially affecting scholastic ability and future employment. Persistent visual and hearing deficits were uncommon ^16^. Poor neurodevelopmental outcome is associated with younger age, delayed presentation and treatment initiation, clinical severity and hydrocephalus ^16, 23, 24^.

As with infarction in adult TBM, childhood TBM-associated infarction commonly occurs in the basal ganglia, damage to which has been associated with language delay, spatial neglect, executive dysfunction, autism, and attention deficit hyperactivity disorder (ADHD) ^25^. Multiple, bilateral, and large infarctions have been associated with worse developmental outcomes in survivors of childhood TBM ^26^. Cognitive deficits can also occur without accompanying physical disability; Schoeman *et al*. described an 80% prevalence of cognitive delay (median IQ 71.5, range 36–102) in children with TBM, with no significant difference in mean IQ between those with and without motor impairment ^16^. In school-age children, up to 43–53% show poor scholastic progress, including grade repetition ^16, 27^.

In a behavioural sub-study of childhood TBM survivors, all had symptoms consistent with ADHD, with similar teacher and parent ratings. The TBM survivors were described as more unpopular, compulsive, and aggressive than their unaffected siblings ^28^. Parents of childhood TBM survivors reported significant social maladjustment and aggression. Mean scores on the Child Behavior Checklist correlated with TBM severity at presentation, indicating emotional disturbance with anxiety, depression, disruptive and rule-breaking behaviour ^28^.

A multidisciplinary approach is required to evaluate and manage the neurodevelopmental sequelae of TBM, which are compounded by low socioeconomic status and limited access to educational support ^15^. Families of TBM survivors experience an increased financial burden as a result of long-term sequelae. One-third of mothers in a South African study had to terminate employment to care for their children, further worsening their precarious socioeconomic situation ^27^.

## Neurocognitive impairment and functional outcomes in infective meningoencephalitis: what can we learn from other infective causes of brain injury?

### Adult

Studies assessing neurocognitive and functional impairment in other infective forms of adult meningitis, although more frequent than for TBM, are still relatively sparse. In a meta-analysis of neurological sequelae post-bacterial meningitis, 61% of studies did not assess cognitive function. Of those where it was assessed, cognitive deficit was one of the most common major sequalae, occurring in 9.1% of people ^29^. Table 1 summarises findings from published studies where cognition and functional measures have been used to assess impairment following infective forms of meningoencephalitis. This summary demonstrates that tools used in these conditions vary widely, and although not exhaustive provides convincing data to support the presence, and the characteristic features of neurocognitive and functional impairment post infective meningoencephalitis. In the studies listed, a minimum of four cognitive domains are tested, most often to include intelligence, memory, executive function and psychomotor function. Assuming that many of the anticipated cognitive deficits seen in TBM have similarities to other forms of meningitis, studies of TBM should include similar neurocognitive measures to those listed within Table 1. However, only one of these studies was performed in a setting where TBM also predominates (Uganda) ^30^ using tests previously validated in sub-Saharan populations ^31, 32^. The use of neuropsychiatric measures such as the Becks depression scale and POMS (profile of mood states) to detect coexistent depressive mood disorders given their likely impact on neurocognitive functioning highlights the need to consider these outcomes in TBM.

**Table 1.** Methods used in selected studies assessing neurocognitive and functional impairment in other causes of adult infective meningoencephalitis.

Reference, study design and aetiology	N	STUDY LOCATION and TIMEPOINT	Neurocognitive assessment *		Functional/ psychological assessment
			Domain	Measure	
Van de Beek *et al.*, JID 2002 ** ^45^ - Prospective cohort - Bacterial	51	Netherlands Median days discharge to testing 391 and 426 in meningococcal and pneumococcal respectively	Intelligence	Groningen Intelligence Test Dutch Adult Reading Test	RAND-36
			Memory	Rey’s Auditory Verbal Learning Test and Wechsler Memory Scale Revised	
			Attention and executive functioning	Trailmaking Test, Stroop Color- Word Test, category fluency, letter fluency, and the Wisconsin Card Sorting Test (WCST)	
			Reaction speed	Simple and 2-choice reaction time measurements	
Hoogman *et al.*, JNNP 2007 ^46^ - Prospective cohort - Bacterial	155	European Dexamethasone Study Time between illness and cognitive testing in months (mean (SD)) 68.8 (meningococcal) and 54.7 (pneumococcal)	Memory	Rey’s Auditory Verbal Learning Test, Rivermead Behavioural Memory Test and Wechsler Memory Scale Revised	RAND-36 POMS
			Attention/executive function	Stroop Test, Groningen Intelligence Test, Trail Making Test part B, Category Fluency, Letter Fluency and Wisconsin Card Sorting Test	
			Psychomotor	Trail Making Test part A Stroop Test, simple and two choice reaction tasks.	
			Intelligence	Groningen Intelligence Test, Dutch Adult Reading Test	
Weisfelt M *et al.*, Ann Neurol 2006 ^47^ - RCT Dexamethasone - Bacterial	87	European Dexamethasone Study Median 99 months between meningitis and testing	Intelligence	Groningen Intelligence Tests, Dutch Adult Reading Test	RAND-36 POMS Grooved Pegboard *
			Memory	Rey’s Auditory Verbal Learning Test River-mead Behavioural Memory Test (RBMT Wechsler Memory Scale-Revised Wechsler Adult Intelligence Scale-Revised	
			Language	Boston Naming Test	
			Attention	Trail Making Test, Stroop Color Word Test, Wechsler Adult Intelligence Scale-Revised Digit Span Test	
			Executive function	Category and Letter fluency and the Wisconsin Card Sorting Test	
			Psychomotor function	Trail Making Test, Stroop Color Word Test, Simple and 2-choice reaction tasks	
Merkelbach *et al.*, Acta Neurol Scand, 2000 ^48^ - Prospective cohort - Bacterial	22	Germany	Intelligence	Multiple Choice Vocabulary Test	BECKS depression inventory
			Memory	Wechsler Adult Intelligence Scale	
			Visual learning and recall	Benton Visual Retention Test	
			Attention and concentration	Aufmerksamkeits Belastungs Test	
			Psychomotor	Number connection test	
Carlson *et al.* Metabol Brain Dis, 2014 ^30^ - Prospective cohort - Cryptococcal	78	Uganda	Verbal learning and memory	World Health Organization- University of California-Los Angeles Auditory Verbal Learning Test	
			Attention and working memory	Digit Span Forward and Backward	
			Language fluency	Semantic Verbal Fluency	
			Speed of information processing, Concentration	WAIS-III Symbol Digit	
			Speed of information processing, Attention	Color Trails 1	
			Executive function	Color Trails 2	
			Timed Gait	Gross Motor	
			Grooved Pegboard	Fine Motor	
			Finger tapping	Motor Speed	
Levine *et al*. J Clin Exp Neuropsychol 2008 ^49^ - Prospective cohort - Cryptococcal, toxoplasmosis encephalitis, progressive multifocal encephalopathy	31	USA	Information processing	Symbol Search, Digit Symbol, Trail Making Test–Form A	Psychiatric Research Interview for Substance and Mental Disorders
			Learning	Hopkins Verbal Learning Test–Revised (Learning Trials 1–3), Brief Visuospatial Memory Test–Revised (Learning Trials 1–3)	
			Memory	Hopkins Verbal Learning Test–Revised (Recall Trial), Brief Visuospatial Memory Test– Revised (Recall Trial)	
			Abstraction	Wisconsin Card Sorting Test– Perseverative Responses, Trail Making Test–Form B	
			Verbal fluency	Controlled Oral Word Association Test	
			Attention/working memory	Letter–Number Sequencing, Paced Auditory Serial Addition Test–Trial 1	
			Psychomotor	Grooved Pegboard (both hands)	
			Visual Memory		
			Cognitive Speed		

*See Table 2 for details and references related to neurocognitive measures ** to delineate whether tests of psychomotor function were impaired due to physical vs cognitive disability POMS, Profile of mood states to detect depressive mood disorders.

HIV can lead to neurocognitive impairment due to chronic sustained immune activation in the central nervous system (CNS) and direct neurotoxicity from the HIV virus and its proteins ^33^. HIV-associated dementia (HAD) is a severe subcortical dementia syndrome associated with significant functional limitation. Prior to widespread use of antiretroviral therapy, HAD was common, occurring in up to 50% of patients prior to death ^34^. HAD is now uncommon in populations with access to effective antiretroviral therapy and is usually associated with treatment failure or undiagnosed advanced disease ^35^. Despite the fall in cases of HAD, milder forms of cognitive impairment persist in the antiretroviral therapy era. This milder impairment has a different phenotype to HAD, with more cortical involvement and executive dysfunction. There are many causes for milder impairment, some of which are directly related to HIV, whereas others relate to comorbid conditions or health related behaviours. In practice, many patients with cognitive impairment often have a combination of factors potentially contributing to their cognitive complaints, and the direct effect of HIV on cognition can be difficult to determine. In TB/HIV coinfected patients it can be difficult to separate TBM-related sequelae from impairment due to HIV and other causes, although the former may be due to focal CNS damage, whereas the latter tends to be diffuse. Obtaining normative values for cognition that are appropriate for the diverse socio-economic backgrounds of HIV-positive populations can be difficult, and there has been controversy about the extent to which current cognitive testing paradigms represent the true prevalence of neurocognitive impairment in HIV-positive populations ^36^. Similar challenges exist obtaining norms for TBM cohorts, particularly as some of the conditions associated with risk of TB and HIV acquisition, such as low socioeconomic status and lack of education, can also be associated with poorer performance on cognitive testing. This highlights the importance of carefully matched, locally derived, normative data.

### Paediatric

Similarly, in children, risk of different long-term complications of postnatally acquired CNS infections is not well-studied despite significant impact on quality of life ^29, 37^. Furthermore, little information is available from resource-constrained settings ^29, 38^. The most common long-term sequelae in CNS infections are cognitive, language, and motor deficits. Studies of neurodevelopmental sequelae have focused on school performance, with few looking at psychopathological impairments ^39^.

In systematic reviews of the risk of disabling sequelae from bacterial meningitis, neurocognitive impairment occurred in 10–25% of children, with a high likelihood of multiple affected domains ^29^. The risk of sequelae in bacterial meningitis has been shown to increase with younger age ^29^ and HIV infection ^40^. Verbal, performance, and full-scale IQ, as well as reading accuracy, comprehension and visuo-motor integration were all significantly lower in school-age bacterial meningitis survivors, compared with age-matched, non-meningitis controls ^41^. In children recovering from bacterial meningitis, even without obvious neurological deficit, there is a risk of long-term cognitive deficit requiring early recognition and management ^42^. In a systematic review of childhood infective encephalitis, 42% had at least one long-term sequela (with a higher proportion (64%) in herpes simplex virus encephalitis). More than one-third suffered from developmental delay, and 10–18% had behavioural impairment, motor deficit, intellectual disability and/or convulsions ^43^.

Epidemiological data describing the impact of disability post-CNS infection on daily life can also influence public health policy. Using the Liverpool Outcome Score tool, researchers of Japanese encephalitis (JE), the most important cause of encephalitis in Asia, demonstrated that 10% of survivors had disability incompatible with independent living. Health staff in Cambodia used these results, along with surveillance and cost-effectiveness data, to support the introduction of a JE immunization programme in 2009 ^44^.

## What outcome measures are available to assess cognitive and functional outcomes in TBM?

### Adult

***Neurocognitive outcomes:*** Targeted assessment of cognitive function in TBM has not been prioritised in most observational and interventional TBM studies. In populations in which TBM is prevalent few screening tests or neurocognitive batteries have been developed or culturally adapted, no normative data is available in order to develop appropriate cut-offs and validation studies have not been performed.

A few studies have used the MMSE, a widely used bedside screening tool for dementia, to evaluate cognitive function in TBM survivors ^3, 4^. The MMSE has lower sensitivity for mild cognitive impairment and may be confounded by age and education. The MMSE can also miss impairment in certain cognitive domains, including executive function. Traditionally, a cut-off score of 24 has been used for possible neurocognitive impairment in clinical practice, although in the two aforementioned TBM studies ^3, 4^ a cut-off of 22 to 29, depending on education, was used to define impairment. The Montreal Cognitive Assessment (MOCA) is a screening tool originally designed to detect mild neurocognitive impairment across multiple cognitive domains (executive function, attention/concentration, and memory) in older adults with Alzheimers Disease ^50^, which has been validated for use in other conditions ^42, 51^, including HIV-cognitive impairment ^52^. Although designed for North American patients, the tool has been validated in other countries such as Japan ^53^, Egypt ^54^ and Korea ^55^. However, in countries where TBM is common, the MOCA has not been validated and floor effects (i.e. some questions are likely to not be answered correctly by all responders) are likely to exist which compromise the appropriateness and usefulness of this screening test. In South Africa, a study to assess the utility of the MOCA in HIV-associated cognitive impairment, floor effects in several domains of the tool were observed suggesting that modifications were required before it could be normed and validated in this population ^52^. 

More in-depth pen-and-paper neuropsychological testing, which is time-consuming and requires a trained examiner, has not been routinely included in observational studies and clinical trials of TBM ^5^. Even if feasibility allows, pen and paper neuropsychological testing like screening tests have similar limitations in populations where TBM predominates; linguistic, cultural and educational differences between these populations and those within the countries where the measures were designed, normed and validated jeopardise the appropriateness of these tests for use in TBM. To overcome this, studies to assess construct validity (the degree to which the measure assesses the cognitive domain in question) in the population of interest needs to be assessed. Table 2 lists measures which can be used to assess deficits in the domains we hypothesise based on the pathophysiology of TBM and are felt to be culturally neutral to ensure appropriateness to the population, and enable future comparison across studies globally.

***Computer-based methods of neurocognitive assessment**:* Computer-based assessments of cognition are an attractive possible alternative to administration of traditional methods of neurocognitive testing by healthcare professionals trained in neuropsychometric techniques. Response and latency times can be measured with greater precision, and the potential for examiner subjectivity is reduced. In resource-limited settings, computer-based methods may be more scalable, as the basic technology required is often more readily available, and less expensive, than neuropsychology expertise. No computer-based cognitive tool has been validated for use in TBM patients specifically; however, the tools discussed below are of potential use.

**Table 2.** Selected neurocognitive outcome measures relating to domains which are likely to be affected in adult tuberculous meningitis and felt to be culturally neutral.

DOMAIN	MEASURES
ATTENTION AND WORKING MEMORY	**Wechsler Adult Intelligence Scale Fourth Edition (WAIS IV)** **Digit Span, Digit Vigilance Test,**
LEARNING AND MEMORY	**Brief Visual Memory Test-Revised (BVMT-R) ^60^**
EXECUTIVE FUNCTION	**Color Trails 2, Design Fluency 1 & 2, Design Fluency Switching**
PSYCHOMOTOR	**Color Trails 1 ^61^, WAIS III ^62^**
VERBAL FLUENCY	**Category Fluency Test, Action fluency**
VISUOSPATIAL	**Judgement of Line Orientation Task**
MOTOR SKILLS	**Grooved Pegboard ^63^, Finger Tapping Test**

Several computer-based tools have been developed to assess cognitive function. Cogstate, which is widely used in HIV research, is designed to be culture and language neutral. It has been shown to function well in clinical trials and is sensitive to subtle impairment and change over time ^56^. However, individual Cogstate tests do not correlate well with domains measured by traditional pen-and-paper methods ^57, 58^. The fact that TBM can lead to focal CNS damage (e.g., from infarction or tuberculoma) may limit the usefulness of Cogstate in this condition. 

The National Institutes for Health (NIH) have developed a comprehensive set of neuro-behavioural measurements. The cognitive module of the NIH Toolbox is more aligned to the construct of traditional pen-and-paper tests and as such has a stronger correlation with domain-specific cognitive function ^56^. This package has a Spanish translation, but otherwise issues with culture and language specificity may limit its use in LMIC. Other computer or phone-based tools aim to provide a screening test or rapid assessment of cognition in a busy clinic. The CAT-rapid, a brief (5-minute) tool on a smartphone app designed for use in resource-limited settings, has been shown to be sensitive for detecting HIV-associated dementia but, as with most screening tools, is insensitive for milder forms of impairment ^59^.

Novel computerised methods for testing certain cognitive domains need to be tested to ensure construct validity (i.e. do the tasks tap into the domain which they are designed to assess?) and subsequently for convergence validity and divergence validity against standard pen and paper tests which test the same and different to the domain in question. For example, in a study of South African HIV-infected adults Katzef *et al.* tested the construct validity of a tablet-based application designed to be culturally fair. In this study, specific measures of processing speed (the swiftness with which one is able to complete mental tasks) were compared to results of equivalent pen and paper tests of the same cognitive domain (convergence validity) with tests of a different domain (divergence validity) ^64^. The use of these emerging computerised tools alongside traditional pen and paper tests in a handful of TBM studies will begin to generate much-needed normative data in the populations of interest, and, in future, pave the way for less resource-heavy standardised methods for testing neurocognitive function in studies of TBM.

***Functional outcomes:*** No one test or battery of tests has been specifically developed or validated for evaluation of functional outcomes in patients with TBM. As a result, functional measures designed for stroke or traumatic brain injury (TBI) patients have been appropriated for use in TBM. The Modified Rankin Scale ^65, 66^ and Barthel Index ^67, 68^, the two most widely used outcome scales in contemporary stroke trials ^69^, are commonly used to assess disability in adult TBM survivors. The Extended Glasgow Outcome Scale ^70^, which has become the standard for measuring functional outcome in individuals with TBI, has been a less popular choice for the assessment of patients with TBM-related brain injury. Other measures, including the Liverpool Outcome Score for children ^71^ and World Health Organization Disability Assessment Schedule 2.0 ( WHO DAS 2.0) may be optimized for TBM patients but have, up to now, not been used in published TBM studies. Benefits and disadvantages of these outcomes, as well as practical considerations for their use in the context of TBM, are discussed below and in Table 3.

**Table 3.** Strengths and limitations of Modified Rankin Scale vs Barthels Index in a tuberculous meningitis setting.

FUNCTIONAL OUTCOME MEASURE	STRENGTHS	LIMITATIONS
MODIFIED RANKIN SCALE (MRS)	• Brevity • Captures impairment in multiple domains (e.g., physical, cognitive, psychiatric) and their impact on overall functional independence • Straightforward interpretation for patients and laypersons • More responsive to change specifically in patients with mild to moderate disability compared with the BI (PMID: 12154262 Weimar)	• High interrater variability due to broad and ill- defined grades; use of a structured interview has been shown to improve interrater reliability (PMID 12215594)
BARTHEL INDEX (BI)	• Ease of administration • Comparable reliability if completed by trained observers or proxy (PMID: 3403500 Collin) • Greater interrater reliability compared with mRS (PMID: 1833860 Wolfe) • Overall more responsive to change compared with mRS and other global measures of function (PMID: 15150715 Dromerick, PMID: 14976324 Kwon)	• May not capture impairment in other domains (e.g., cognitive, psychiatric) that are often impacted in TBM survivors due to its focus on ADLs and physical impairment • Limited ability to discriminate among higher functioning individuals due to “ceiling effect,” in which large proportion of patients achieve maximum possible score (PMID: 21372310 Quinn, PMID: 15150715 Dromerick)

***Modified Rankin Scale:*** The modified Rankin Scale (mRS) is a clinician-reported outcome scale with 6 grades ranging from no symptoms to severe disability requiring 24-hour care ^65^. The mRS is a global disability rating scale measuring overall functional independence, considering performance of basic and instrumental activities of daily living (ADL) which prompts the clinician to consider the impact of impairment in multiple areas (e.g., physical, cognitive, psychiatric) on perceived disability. Transitions between the different mRS grades are considered to be clinically meaningful ^72, 73^ and correlate well with patient-reported outcomes ^74^. Although originally developed to characterize recovery after stroke ^66^, the mRS has been used to assess functional outcomes in other conditions, including meningoencephalitis ^75, 76^. The mRS has good validity, at least among stroke survivors in whom most studies assessing its psychometric properties have been performed ^77^. The main criticism of the mRS is that the grades are too broad and ill-defined ^65^, resulting in high interrater variability. Use of a structured interview and assessor training may reduce bias and improve interrater reliability ^70, 73, 77^.

The mRS has been used in several observational and interventional studies to assess functional outcome in TBM ^78– 83^. In a large, randomized controlled Vietnamese trial of dexamethasone versus placebo for TBM, the mRS was trichotomized as: grade 0 indicating a “good” outcome, grades 1–2 indicating an “intermediate” outcome, and grades 3–5 indicating “severe disability.” In the trial, 38% of participants who received dexamethasone had a good outcome compared with 35% who received placebo. No consensus exists regarding the optimal mRS cut-off to define favourable versus unfavourable outcomes, which is a major barrier to combining data across trials. Some TBM studies have classified grade 3 (moderately disabled) as an unfavourable outcome, whereas others consider grade 3 to be favourable ^78, 82, 84^.

Careful definition of an endpoint using the mRS is essential and can make the difference between a significant and non-significant result ^85^. The reductionist approach of dichotomizing the mRS may fail to detect improvement in function by one grade, particularly among individuals with minimal or severe disability. For example, if a patient’s mRS grade improves from 1 to 0 or worsens from 4 to 5, this clinically relevant information is not captured when the mRS is dichotomized. An alternative to using the mRS as a dichotomous outcome is to evaluate the entire distribution of grades via a shift analysis ^72, 73^, which may offer a more nuanced view of the effect of an intervention (see Box 1).

**Box 1. Benefits and limitations of using shift analysis for the Modified Rankin Scale (mRS) in clinical trials**

PROS

- Avoids loss of clinically meaningful information that occurs with dichotomizing the mRS outcome
- Potential to detect smaller treatment effects compared with dicohotomous approach
- May improve power and statistical efficiency ^86^

CONS

- Assumes that treatment effects of an intervention are uniform across the range of mRS grades ^73^
- May actually reduce power compared with dichomotous approach if misclassification errors are not uniform across the range of mRS grades ^73^
- May be less reliable in trials with small patient samples

Definition of shift analysis: Analytic approach to the mRS gaining traction in stroke research ^87^ that exploits the full distribution of possible mRS outcomes by assessing its entire ordinal scale range (as opposed to dichotomization).

*Barthel Index:* In contrast to the mRS, which is a global measure of function, the 10-item Barthel Index (BI) has a narrower scope that focuses on physical constructs and independence in basic ADL. The original BI, scored out of 100, was developed to evaluate disability in patients with stroke and other disorders ^67^. The modified BI ^68^) captures the same content but employs a different scoring system ranging from 0 to 20. The BI has good to excellent internal consistency, test-retest, and interrater reliability ^69, 88, 89^.

Although the mRS and BI are highly correlated ^90^, each has strengths and limitations that should be taken into account when selecting the best measure for an observational study or interventional trial ( Table 3). Like the mRS, no consensus exists on the optimal cut-off to distinguish a favourable from unfavourable outcome on the BI. Studies have identified a BI score >90 or >80 (>18 and >16, respectively, on the modified BI) as the critical threshold for independence ^91– 93^. A score of <60 on the BI (<12 on the modified BI, is often used to indicate a poor outcome ^87^.

The modified BI has been used in both observational studies and interventional trials in TBM ^4, 94, 95^. In a retrospective study evaluating predictors of functional outcome at one year in 65 adults in India with TBM using the modified BI, 51% had no limitations in ADLs, whereas 43% had poor recovery (score<12) ^4^. These results were similar to a Turkish study using the same cut-offs to assess functional outcomes at one year in 61 adults with TBM. Among the 44 survivors, 41% recovered completely compared with 16% with poor recovery ^95^. In two aforementioned interventional trials the modified BI was used to assess functional outcome ^94, 96^. No significant difference in functional outcome was detected between groups.

*Extended Glasgow Outcome Scale:* The Glasgow Outcome Scale (GOS) is the prevailing functional outcome measure in TBI trials. The original GOS is an ordinal scale with five categories: death, vegetative state, severe disability, moderate disability, and good recovery. In the GOS-Extended (GOSE), the last 3 categories are further divided to improve detection of smaller, clinically meaningful changes ^70^. The application of the GOS(E) in TBM studies has been limited ^1, 97^. The open-ended format of the GOS(E) can result in substantial interrater variability. Like the mRS, use of a structured interview and assessor training improves interrater reliability ^70^.

*WHO Disability Assessment Schedule (DAS) 2.0:* The WHO DAS 2.0 is a generic instrument that assesses health and disability based on the conceptual framework of the WHO International Classification of Functioning, Disability and Health (ICF) ^98^. The WHO-ICF categorizes function in terms of impairment, activity, and participation. Unlike the mRS and BI, which each only address one dimension of the WHO-ICF (participation and activity, respectively), the DAS 2.0 captures information about all three dimensions. The standardized instrument, which was developed for use across cultural contexts and has been translated into 27 languages, evaluates six domains: cognition, mobility, self-care, getting along, life activities, and participation ^99^. The DAS 2.0 has high internal consistency, test-retest reliability, and good validity ^99^. Although it has been validated for use in TBI and other brain injuries, no studies have used it to assess functional outcomes in TBM.

### Paediatrics

Numerous instruments have been developed for the assessment of child development based on age, domains tested, and type (performance based, self/caregiver rating). Unlike in adult TBM, these have been extensively reviewed elsewhere in published literature including comparison of available tests and suitability for use in LMIC settings ^100, 101^. Although there is no consensus or consistency on the measures used to assess outcome post TBM in infants and children, ideal testing requires wide age-range; the ability to measure floor and ceiling effects as some of the children are left with very low residual function, and needs to assess fine and gross motor ability, receptive and expressive language as well as behavioural, social and adaptive skills. We have made recommendations for testing in paediatric TBM in Table 4.

**Table 4.** Recommendations for testing neurocognitive and functional impairment in tuberculous meningitis studies.

POPULATION	OUTCOME	MEASURE	TIMING
**ADULT**	**NEUROCOGNITIVE** *Trained individual required for test administration	**MINIMAL:** Montreal Cognitive Assessment (MOCA) using where possible locally derived data to ascertain suitable cut offs for testing population	‘Post-acute’ (6–9 months)
		**OPTIMAL:** 1. Neurocognitive battery administered in patients first language using culturally neutral measures. as listed in Table 2 2. Measures of potential confounders to cognitive testing – eg. depression, drug use, traumatic brain injury, educational background. 3. Collection of data in well-matched healthy controls to obtain ‘normative’ population data 4. Validation of computerised tests to assess corresponding domains	Initially ‘post-acute’ assessment at 6–9 months *plus* long term follow up at 2 years
	**FUNCTIONAL**	**MINIMAL:** Modified Rankin Scale +/- Barthel Index (see Table 3 on suitability of measure to study design)	Post-acute (6–9 months)
		**OPTIMAL:** Modified Rankin Scale +/- Barthel Index (see Table 3 on suitability of measure to study design) plus additional World Health Organization Disability Assessment Schedule 2.0	Post-acute (6–9 months) and long term follow up
PAEDIATRIC **	**NEURODEVELOPMENTAL** Assessing motor (gross and fine) language, social, reasoning/behaviour +/- vision, hearing. *Specialist training required for test administration	**MINIMAL:** Ages and Stages Questionnaire ^102^ Used in many settings Age range: 0–5 years [Adaptation to local language, collection of data in matched healthy controls to obtain ‘normative’ population data.]	Post-acute – 6–9 months, 2 years and 4 years (age allowing)
		**OPTIMAL:** *Bailey Scale of Infant Development – Third Edition ^103^ Age range 1–42 months *Mullen Scale Early Learning ^104^ Age range: 0–68 months [Adaptation to local language, collection of data in matched healthy controls to obtain ‘normative’ population data]	
	**NEUROCOGNITIVE** *Specialist training and license required for test administration	*Weschler Intelligence Scales for Children ^105^ Tested and used in many settings but validity not well established in LMIC Age range: 6–16 years Kauffman Assessment Battery for Children- second edition ^106^ Age range: 3–18 years [Adaptation to local language, collection of data in matched healthy controls to obtain ‘normative’ population data.]	Post-acute (6–9 months) and long term follow-up throughout schooling (eg 2 and 5 years minimum)
	**FUNCTIONAL**	**MINIMAL**: Modified Rankin Scale (mRS) Age range: 1 year to adult **OPTIMAL:** Vineland Adaptive Behaviour Scale ^107^ Age range: birth – 18 years	Post-acute (6–9 months) and long term follow-up throughout schooling (eg 2 and 5 years minimum)
	**NEUROBEHAVIOURAL**	**MINIMAL:** Strengths and Difficulties Questionnaire ( http://www.sdqinfo.com) Age range: 4–17 years Child, parent, teacher forms [Adaptation to local language, collection of data in matched healthy controls to obtain ‘normative’ population data.]	Post-acute (6–9 months) and long term follow-up throughout schooling (eg 2 and 5 years minimum)

**The above developmental assessment tools have not been formally adapted for use in LMIC nor have locally determined norms been developed. Therefore, interpretation of results requires careful consideration. It is acknowledged that a number of locally developed screening tools, not detailed above, are available for use in specific country settings.

## What are the knowledge gaps?

The physical and cognitive disabilities observed in meningitis have long term socioeconomic implications ^108^; however, the extent of this in TBM specifically is unknown. In addition, capturing data on patient-centred outcomes, including mood and quality of life, is essential to understanding and intervening on the impact that TBM has on health from the patient perspective. Qualitative research on patients’ and families’ perspectives on the disease and potential barriers to recovery and reintegration is lacking.

Childhood neurodisability is one of the most important precursors of psychopathology, poor adaptive functioning and educational disadvantage. In later life those affected are less likely to be living independently, be in paid employment or have cohabiting relationships compared with controls ^109^. There is renewed global commitment to the improvement of early child development outcomes, as evidenced by the focus of the United Nations Sustainable Development Goal (SDG) 4. SDG4 highlights the need for reliable, valid measures to evaluate preventive and interventional efforts designed to affect change and mandates the systematic monitoring of the health and well-being of all children to achieve optimal development ^110^.

While it is known that TBM causes significant neurocognitive, neurodevelopmental and functional impairment in children and adults, there are limited global epidemiological data using well-validated assessment tools that document findings across different ages and populations. To understand and characterise impairment requires appropriately developed tools to assess neurodevelopment, cognition, and functional outcomes for early identification and treatment of disability and to improve opportunities for developmental change and rehabilitation ^111^.

The major challenge, however, is the paucity of robust and standardised assessment measures, developed for and normed across different geographical and cultural settings. Most published data have used a wide range of tools developed for high income countries (HIC). While tests may be translated into local languages, this is often without validation using local norms or adaptation to local culture. Applying Western-based norms to LMIC carries the risk of identifying healthy children as delayed, and adults as cognitively impaired. For example, measures of non-verbal ability in HICs may not evaluate the same construct across cultures such that results cannot be interpreted in the same manner ^112^. The absence of local or country-specific normed data results in use of statistical strategies to standardise test scores by age ^113^. However, a number of researchers have begun to address this challenge in the paediatric population. In a study of rural Beninese children, Bodeau-Livinec *et al.* evaluated the construct validity of comparing both raw scores and HIC-based standardised scores for two different assessment tools, the Mullen Scales of Early Learning and Kaufman Assessment Battery for Children ^113^. Their findings support the use of a local comparison group to allow for adjusted raw score comparisons. Others have reviewed the suitability of well-established neurodevelopmental assessment tests (NDAT) for adaptation to LMIC ^114^. Gladstone took a different approach with the Malawi Developmental Assessment Tool, producing a culturally relevant NDAT with age-standardised norms for Malawian children ^115^. More recently, a culturally neutral, caregiver report tool used to monitor pre-school children across multiple LMIC settings has been published. Feasibility testing and piloting across a number of LMIC are planned ^116^. To fully understand the burden of neurodevelopmental impairment caused by TBM will therefore, require appropriately adapted, as well as new, locally developed measures of neurodevelopment to detect both early developmental and later, emergent speech, behavioral and cognitive difficulties.

## Future recommendations

The need to describe better the incidence, severity and character of neurocognitive and functional impairment in TBM is clear, particularly in adults. Given the low-income settings in which TBM predominates, standardised, locally normed functional and neurocognitive assessments that do not require costly or extensive staff are needed. In children these need to vary by age targeting early developmental skills (e.g., language, motor, and visual-receptive) in children under 5, and more domain specific assessment (e.g., attention, emerging executive functioning) in older children. Measurement of emotional and behavioural functioning in the daily setting from complementary parent/teacher sources is also necessary. In adults, these should in the first instance endeavour to estimate degree of cognitive impairment across multiple domains which we anticipate will be most impaired in TBM. Computer-based tests provide the obvious solution; however, construct validity and (lack of) normative data sets limit their use. Whilst these are in development a uniform method for adapting currently available assessment tools, including translation/back-translation of instructions into local languages or adjusting stimuli for cultural differences, is necessary. Further, prospective studies should include a control group for confounding variables (e.g., socioeconomic status, local culture/customs).

To date, no research into long-term outcomes in childhood TBM survivors has evaluated interventions on daily functioning, and therefore measures of adaptive function should be included. Similarly, in adults, the few studies of cognitive and functional impairment post TBM do not consider longer term sequelae in TBM. A recently published study suggested pathways associated with glutamate neurotransmitter release, NMDA-receptor binding and GABA degeneration may play a role in TBM pathogenesis ^117^. Given that these neurotransmitters are also implicated in the pathogenesis of neurodegenerative conditions such as Alzheimer’s Disease ^118, 119^, there is an increasing need to describe the clinical manifestations of long-term sequalae in TBM.

Based on currently available data we have comprised a table of recommendations for testing neurocognitive and functional outcomes in adults and children with TBM ( Table 4). We recommend that investigators designing clinical studies in TBM consider integrating these measures as part of clinical follow-up.

This table includes pragmatic approaches to assessing deficit in low and high functioning individuals. It suggests intervals for testing, including timing of long-term follow up which, where feasible, should be considered in TBM studies. We suggest that efforts to validate computerised methods for cognitive testing in our populations of interest need to be made in order to feasibly develop neurocognitive assessment as a viable biomarker of clinical outcome in the future.

Unlike in adult TBM, paediatric research in this field is sufficient to rationalise a pragmatic treatment approach. We recommend that this should involve rehabilitation (e.g., speech-language, occupational and physical therapies) with consideration for pharmacological intervention (e.g., stimulants for inattention). Early treatment initiation affects functional outcomes in other forms of brain injury ^120^ and therefore where possible these should be offered in the acute phase of TBM. Additionally, post-acute and long-term care studies in these populations demonstrate ongoing need for rehabilitative and psychosocial intervention and, where feasible, this should be considered. Behavioural dysregulation is common in childhood CNS disease; for example, studies in HIV-infected children suggest that interventions targeting caregiver behavioural management improves cognition and behaviour ^121, 122^; similar approaches need to be tested in TBM. Universal lessons from other CNS infections indicate that prospective longitudinal studies focused on quality of life and improving health, vocational, and socioemotional adjustment are needed. Collecting high-quality data that demonstrate the disease’s impact will further support advocacy for improved TB prevention programmes and is required to assess the resource burden and effectiveness of treatment interventions ^123^.

## Data availability

No data are associated with this study.
