# Supplementary material for: Neurocognitive and functional impairment in adult and paediatric tuberculous meningitis
Source: Wellcome Open Res. 2019 Nov 13;4:178. [Version 1] doi: 10.12688/wellcomeopenres.15516.1 (PMC6971841; doi:10.12688/wellcomeopenres.15516.1)
Supplement: Peer review history

# Peer review: Reviewer response for version 1

**DOI:** 10.21956/wellcomeopenres.16984.r37378

**Published:** 2020-01-20

**Authors:** Monica Marta

**Affiliations:** 1 Blizard Institute, Queen Mary University of London, London, UK; 2 Barts Health NHS Trust, London, UK

This extensive review of the state of the art about neurocognitive impairment in TBM is well written, extensively based on published data and includes tables that highlight important messages. This review is a useful tool to chose cognitive tests for a future study or even in clinical practice.

 Despite not being part of the subject of the review, it would be important to highlight how difficult it is to have a clear diagnosis of TBM both in resource-scarce and even in state of the art centres. It is not clear from the review how certain alternative and co-diagnoses may confound the outcomes: HIV is described but other infections such as malaria or bacterial meningitis are not.

 Furthermore, drugs other than anti-TB are not assessed: e.g. the use of anti-epileptic medication and which drugs are used can be a confounder in the neurocognitive measurements.

 If possible, it would be interesting to read about which aspects of neurocognitive impairment the authors consider amenable to rehabilitation.

 I am aware my questions are beyond the scope of this review, but maybe a mention in the Discussion would be useful.

I confirm that I have read this submission and believe that I have an appropriate level of expertise to confirm that it is of an acceptable scientific standard.

# Peer review: Reviewer response for version 1

**DOI:** 10.21956/wellcomeopenres.16984.r37049

**Published:** 2019-12-20

**Authors:** Reuben Robbins

**Affiliations:** 1 HIV Center for Clinical and Behavioral Studies, New York State Psychiatric Institute, New York City, NY, USA

This is a well-written and thorough review of the neurocognitive and functional sequelae of tuberculous meningitis (TBM) – a mostly understudied area. There are a few minor points that could be revised.

 First, the introduction is fully without any citations – many statements are made without support. However, the other sections of the manuscript provide relevant support for the statements made in the introduction.

 Second, the idea that neurocognitive and functional tests need specific adapting/validating for TBM is not totally correct. Most neurocognitive and functional tests were designed to assesses general abilities, irrespective of disease. What I think the authors are trying to say is that with such little research in this area, it is very difficult to know which or what types of these tests are best suited to detect the cognitive and functional sequelae of TBM. They provide useful recommendations that address this gap in the literature, but seem to suggest that tests need specific validation beyond robust cultural adaptions/translations and appropriate normative data collection.

 Third, the issues of culturally validity and proper norms are not unique to HIV research, but rather speak to the field of doing these types of assessments across any disease.

 Fourth, “Becks depression scale” should be changed to either “the Beck Depression Inventory” or “Beck’s depression scale.”

 Finally, regarding novel computerized methods of neurocognitive assessment, the authors refer to the Katzef et al. article; they may also consider citing Robbins et al. (2018) ^1^.

I confirm that I have read this submission and believe that I have an appropriate level of expertise to confirm that it is of an acceptable scientific standard.

## References

[1] : A Mobile App to Screen for Neurocognitive Impairment: Preliminary Validation of NeuroScreen Among HIV-Infected South African Adults. *JMIR Mhealth Uhealth*.2018;6(1) : 10.2196/mhealth.9148 e5 10.2196/mhealth.9148 29305338PMC5775487
